# Assessment of Blood Donation and Transfusion in Eastern Uganda: A Mixed-Methods Study

**DOI:** 10.5334/aogh.2426

**Published:** 2019-04-15

**Authors:** Laura Checkley, Girish Motwani, Irma Catherine Wange, Obieze Nwanna-Nzewunwa, Fred Kirya, Mary Margaret Ajiko, Catherine Juillard, Rochelle A. Dicker

**Affiliations:** 1 Center for Global Surgical Studies, Department of Surgery, University of California, Zuckerberg San Francisco General Hospital & Trauma Center, San Francisco, US; 2 Department of Surgery, Soroti Regional Referral Hospital, Soroti, UG; 3 Department of Surgery, University of California, UCLA Surg-Gen, Los Angeles, US

## Abstract

**Background:**

Blood and blood products are essential in the management of injuries, medical illnesses, and childbirth. Chronic shortages in the blood supply perpetuates the high levels of morbidity and mortality from injury and treatable diseases. Patients in low- and middle-income countries are frequently unable to access blood units necessary for transfusion in a timely manner.

**Objectives:**

This study aimed to gain insight into the community and hospital factors that contribute to the observed insufficient supply of blood units available for transfusion at a regional referral hospital in rural Eastern Uganda.

**Methods:**

A mixed-methods approach was utilized; community members were surveyed on knowledge, attitudes, and practices of blood donation and health professionals were queried on hospital factors affecting blood transfusions. Transfusion records were prospectively collected and analyzed, and the pathway of a single blood unit was observed and recorded.

**Findings:**

Among the 82 community members that were surveyed, knowledge was poor (<50% correct) regarding age, weight, and volume of blood to be able to donate, but participants were overall knowledgeable on general characteristics that would exclude individuals from donating blood. Major themes elicited during qualitative interviews included a positive attitude towards and lack of information regarding blood donation. Health professionals expressed frustration in delayed testing of transfusion transmissible infections. The majority of blood transfusions were allocated to female patients (55.8%) and children under five years of age (33.2%).

**Conclusions:**

Broadened inclusion and education of the general population in blood donation and increased outreach programs may be promising interventions to increase the blood supply at the Soroti Regional Referral Hospital. To reduce the current bottleneck seen in TTI testing, the feasibility and cost-effectiveness of local TTI testing technology should be investigated further.

## Introduction

Maintaining a safe and adequate blood supply is crucial to ensuring positive outcomes of patients in both emergent and non-emergent situations. Low- and middle-income countries (LMICs) frequently have insufficient blood supplies to meet demand. In 2006, only 41.5% of the demand for blood in the World Health Organization (WHO) African Region was met [1]. Of the approximately 112.5 million blood units collected worldwide annually, 50% are collected in high-income countries (HICs), which comprises only 19% of the world’s population [2].

The WHO recommends voluntary, non-remunerated blood donation (VNRBD) and has set a standard of 10 blood donations/1000 population as a baseline value for all countries to meet [2]. While on average HICs have 32.1 donations/1000 population, LICs have only 4.6 donations/1000 population. It is estimated that blood donation by only 1% of a country’s population is needed to meet the basic demand for blood [3]; to achieve this, the WHO advises that all activities related to blood donation, including collecting, testing, processing, and storage, be centralized at the national level. While many countries strive to achieve this, it can be more difficult for LMICs with poor infrastructure and lower healthcare funding.

Common barriers in LMICs, both with blood donation and processing of blood units, make it difficult to maintain an adequate blood supply [4]. A 2017 review by Asamoah-Akuoko et al. of 35 studies on the motivations and deterrents to blood donation in 16 Sub-Saharan African countries found fear as a major deterrent, including fear of needles, adverse effects, and discouraging spiritual, religious, and cultural perceptions of blood donation [5]. Given the unique cultural and societal factors that may affect one’s perception and knowledge of blood donation, community-specific education is essential to recruiting a steady donor pool.

Once a blood unit is acquired, it must undergo appropriate processing before it can be transfused into a patient. One notable logistical barrier is testing for transfusion transmissible infections (TTIs), which is often expensive and time-consuming, particularly for rural hospitals. The four TTIs that the WHO recommends be screened for are HIV, Hepatitis B and C, and Syphilis; LMICs have a proportionally higher prevalence of TTIs compared with HICs [2], stressing the importance of accurate testing.

Uganda is an East African LIC with nearly 20% of its population living below the poverty line [8]. While Uganda has a national blood transfusion service, there has been a noticeable lack of blood available for patients at Soroti Regional Referral Hospital (SRRH), a 300-bed teaching hospital serving the northeastern region of Uganda, roughly a population of 2 million people [9].

While a number of knowledge, attitude, and practice studies have been carried out in specific African populations, few have used qualitative methods to allow for unbiased assessment of perceptions towards blood donation both within the general population and healthcare professionals. The objective of this study was to gain a greater understanding of the community and hospital factors that contribute to the observed insufficient supply of blood units available for transfusion at SRRH. A mixed-methods study was conducted to: (1) understand community members’ knowledge of, attitudes towards, and practice of blood donation; (2) understand SRRH-based providers’ attitudes towards and practices of blood transfusion; (3) describe the distribution of patients receiving blood products; and (4) determine the typical path a single blood unit takes from donation to transfusion in Soroti.

## Methods

### Community Members

Interviews were conducted with community members within Soroti District. Participants were selected via convenience sampling from various parts of the community including schools, businesses, the market, and farming villages. The interview contained two parts: one that sought to assess the knowledge of blood donation and one that sought to understand the attitudes towards and practices of blood donation. Basic demographic information was obtained prior to the start of the interviews. Interviews were conducted in the language of preference (English or Ateso) with the help of a local research assistant.

*Knowledge subsection*: We developed a short questionnaire based off a 2016 study in Gondar Town, Ethiopia, containing five multiple choice questions and five yes/no questions [7]. The questionnaire centered around basic knowledge of blood donation (questions are detailed in Table 2). To remain consistent with the 2016 Gondar Town study, getting >50% of questions correct was considered a passing score on the assessment. Participant donation practices were also queried. Microsoft Excel 2016 was used to manage the data and Stata 15 was used for basic statistical analysis. Chi-square or Fisher’s exact tests and Kruskal-Wallis tests were performed on categorical and continuous variables, respectively. A p-value of 0.05 or less was chosen to indicate statistical significance.

*Attitudes and practices subsection*: For a subset of participants, the interview extended to include the attitudes and practice subsection in addition to the knowledge questionnaire. The semi-structured interview contained a total of 11 open-ended questions on a variety of topics regarding blood donation. Interviews were digitally recorded and later transcribed. Atlas.ti 1.6.0 was used to code and manage the data. Grounded theory was used to generate themes from the content of the dialogues. Two researchers independently developed codes while analyzing the same three transcripts and based on areas of divergence and agreement, synthesized a rough coding outline. Themes were extracted based on final coding schemes and frequency of foci explored by participants.

### Health Professionals

Semi-structured qualitative interviews with health professionals were conducted in English at SRRH. Participants were recruited via convenience sampling and included nurses, attending physicians, and intern doctors from any specialty. Six questions were asked regarding resource allocation, workarounds to address low blood availability, and specific improvements to the blood transfusion/donation process. Qualitative analysis was carried out the same as the community member attitudes and practices subsection.

### Blood Transfusion Records

Demographic data of blood transfusion recipients was prospectively collected from June 2017 to August 2017. Data was managed using Microsoft Excel 2016 and analyzed to characterize utilization trends of different populations receiving blood transfusions.

### Process Mapping

All steps of blood donation and transfusion from procurement of blood to disbursement of blood units within the hospital were observed and recorded. Observation of health workers included field workers collecting blood, lab technicians at SRRH storing and discarding blood, and lab technicians at the Mbale Regional Referral Hospital screening for TTIs. Observations were then synthesized into a process map.

### Ethical Considerations

This study was approved by the University of California, San Francisco Office of Ethics and Compliance, Human Research Protection Program, and Institutional Review Board as well as SRRH. Written consent was obtained from all community members and verbal consent was obtained from all health professionals.

## Results

### Community Members

A total of 82 community members were surveyed. Approximately half were male (51%) and Non-Catholic Christian (51.2%), and the majority had a secondary education or higher (83%; Table 1). They ranged from 17–70 years (median = 28.5 years).

**Table 1:** Demographic data of quantitative community member interviewees.

Characteristics	Frequency (n = 82)	Percentage (%)

Sex		
Male	42	51.85
Female	40	49.38
Education		
Primary	14	17.07
Secondary	40	48.78
Tertiary	28	34.15
Age		
Median (IQR)	28.5 (21–41.75)	–
Relationship Status		
Single	32	39.02
Married	45	54.88
Other (religious worker, divorced)	5	6.10
Occupation		
Secondary school student	17	20.73
Tertiary school student	7	8.54
Farmer	16	19.51
Small business owner	10	12.20
Private employee	20	24.39
Government employee	8	9.76
Religious worker	4	4.88
Religion		
Non-Catholic Christian	42	51.22
Catholic	32	39.02
Islamic	3	3.66
Other	5	6.10

IQR: interquartile range.

*Knowledge subsection*: Less than 50% of participants correctly identified the minimum age and weight eligible for blood donation and maximum volume of blood that a person can donate at once (Table 2). Most, however, knew the appropriate frequency a person can donate blood and what the preferred blood type to have is. Participants also largely understood that pregnant, menstruating, and breastfeeding women and diabetics could not donate blood. However, there was less knowledge that smoking alone does not prohibit one from donating blood.

**Table 2:** Knowledge of community members regarding basic blood donation requirements.

Type of Question	Question	Correct (%)	Wrong (%)

Multiple Choice			
	What is the minimum age eligible for blood donation?	4.88	95.12
	What is the minimum weight eligible for blood donation?	36.59	63.41
	How often can a person donate blood?	69.51	30.49
	Is there a best blood type for donors? What is it?	54.22	45.78
	What is the maximum volume of blood that one person can donate at one time?	18.29	81.71
Yes/No			
	Can pregnant women donate blood?	96.34	3.66
	Can women who are menstruating donate blood?	84.15	15.85
	Can breastfeeding mothers donate blood?	85.37	14.63
	Can people with diabetes donate blood?	90.24	9.76
	Can people who smoke donate blood?	32.93	67.07

Having an education level of tertiary school was associated with a higher chance of passing (>50% of questions answered correctly), while being a student correlated with failing compared to the rest of the population (p = 0.024, 0.046, respectively; Table 3). Thirty-five participants (42.7%) had donated at least once in their lifetime.

**Table 3:** Demographic characteristics associated with knowledge regarding blood donation.

Characteristic	Pass (%)	Fail (%)	p-value

Sex			
Male	71.43	28.57	0.788
Female	68.89	31.11	
Education			
Primary, Secondary	63.08	36.92	0.024*
Tertiary	86.21	13.79	
Age			
Median	30.5	24.00	0.114
Relationship Status			
Single	59.46	40.54	0.106
Married, Divorced	75.47	24.53	
Occupation			
Student	54.17	45.83	0.046*
Farmer	66.67	33.33	0.717
Small business owner	78.95	21.05	0.351
Private employee	66.67	33.33	0.687
Government employee	100	0.00	0.052
Religious worker	100	0.00	0.183

P-values < 0.05 (*) were considered statistically significant.

*Attitudes and practices subsection*: Theoretical saturation was reached after 24 interviews. Characteristics of community member participants are shown in Table 4. Five prominent categories were identified from which major themes were elicited (Table 5): (1) attitudes towards blood donation; (2) motivations of blood donation; (3) deterrents of blood donation;

(4) blood donation safety; and (5) lack of information.

**Table 4:** Demographic features of qualitative subjects.

Characteristic	Community Members (n = 24)	Health Professionals (n = 20)

Age (median)	17–66 (31)	23–47 (30.5)
Sex		
Male	11	5
Female	13	15
Profession (n)	Farmer (6)	Nurse (2)
	Secondary School Student (5)	Intern Doctor (14)
	Tertiary School Student (4)	Attending Physician (4)
	Small Business Owner (4)	
	Business Employee (4)	
	Religious Worker (1)	
Experience	–	<1–22 years*

* Intern doctors all had experience of <1 year, while the other health professionals ranged from 3–22 years.

**Table 5:** Key themes elicited from community member qualitative interviews.

Category	Theme	Excerpt

Attitudes towards Blood Donation	Positive Attitude	“… blood donation is very good for people’s health, like in the main hospital, we can use [it] for those ones who don’t have blood… it increases the lifespan of someone.” [14]
Motivations of Blood Donation	Altruism, Obtain Blood in the Future	“…do this [donate blood] in order to save someone’s life. Like tomorrow, you donate and maybe your mother will fall sick. You will show your card you used for donating blood and your mother will be given some blood.” [34]
Deterrents towards Blood Donation	Lack of Food Security, Sickness	“No food because us students do [not have] cash. So the small [money] we have, we have to use it for other things. So someone who has donated blood has to eat a lot of greens, fruits…sometimes you can’t get.” [34]
Blood Donation Safety	Safe Blood Donation Procedures	“I don’t think I can get any diseases in the blood donation process because every person who goes to donate blood, they get a new needle to get their blood, unless maybe the person who is doing the blood donation process has an intention of infecting… But the process of blood donation is clean and safe because everyone gets a new needle and once they are done donating, it is disposed…” [46]
Low Community Donation Rates	Lack of Information and Community Outreach Programs	“Yes, given an opportunity…one can go and donate, but going to the hospital is what is hard. If they could bring those outreaches, maybe to the market, communities…” [37]

The specific participant’s study identification number is represented in brackets.

Participants displayed an overall positive attitude towards blood donation and the majority were willing to receive a blood transfusion if medically indicated. The key motivations for donating blood were to save lives and to be able to receive blood in the future for oneself or a family member. Participants held a widespread belief that donating blood allows for higher priority of the donator and family members to receive blood in future times of need. Lack of food security and sickness were mentioned as deterrents from blood donation. Participants who cited poor feeding often could not afford the food they thought was necessary to sustain a healthy physique. Respondents had an extensive understanding of donation procedures, acknowledging that trained staff carry out the procurement of blood and use sterile materials, minimizing the chance of infection. Lastly, participants consistently expressed that there is limited information regarding how and where to donate blood as well as few community-based blood donation centers and outreach programs. Multiple people advocated for an increased outreach presence.

### Health Professionals

Theoretical saturation was reached at interview 20. Characteristics of health professional participants are reported in Table 4. Four salient categories emerged from which multiple themes were extracted (Table 6): (1) transfusion transmissible infections (TTIs); (2) indications for transfusion; (3) criteria prioritizing patient populations; and (4) referral to larger hospitals.

**Table 6:** Key themes elicited from health professional qualitative interviews.

Category	Theme	Excerpt

Transfusion Transmissible Infections	Prolonged Screening	“…we had numbers of patients, most of them with [hemoglobin] below 5, and they needed blood, but there was no blood in the hospital. Most of the blood was not screened, and the screening system from Mbale already failed and we were now sending our samples to Nakesero Kampala. We even lost some patients…” [8]
Indications for Transfusion	Clinical Features: Pallor, Acute Bleeding	“Most times we just do it clinically because most of the patients we receive them late in the night…if we see a patient is really paper white…we just take off a sample of blood to do our investigations the next day, but then we go ahead and transfuse these patients.” [11]
	Clinical History: Sickle Cell Disease	“So history you will be able to know if this patient is a known sickler…if it is a sickler, history of already known sickler, the chances of transfusing this patient are already increased.” [6]
Prioritizing Patient Populations	Highest Priority: Children, Pregnant Mothers; Lowest Priority: Elderly, Patients with Terminal Illnesses	“…occasionally [I] look at who is likely to benefit the blood transfusion more …[you] have a woman who is severely pale with severe malignancy. Then you have a baby who is severely pale from malaria. Benefits. I give blood here to this baby, she is likely to benefit from it. Someone who has a malignant process… probably their prognosis is poor…” [12]
Absence of Blood Units Available for Transfusion	Referral to Larger Hospital	“…we contact the head of the blood bank in Soroti, ask them how soon do we hope to get blood [from Mbale]. If it is not within that day, then we shall request the mother to look for funds and take the child to Mbale where they can do the transfusion.” [4]

The specific participant’s study identification number is represented in brackets.

Health professional participants cited significant challenges to screening the four required TTIs. They noted that samples must be sent to the closest machines available for testing, which are located at the Mbale Regional Referral Hospital (MRRH), approximately 100 kilometers away from Soroti. However, during the time of this study, the machines at MRRH were frequently out of order, causing samples to be rerouted to the Nakasero National Blood Bank, roughly 300 kilometers away. Because units were not screened, they remained unavailable for transfusion. The use of clinical features such as skin pallor or the presence of acute bleeding are the most common identifiers that participants used to determine when to transfuse a patient. Laboratory testing such as hemoglobin levels and complete blood counts are available at SRRH, but participants noted that they are often not accessible or practical for them to rely on due to laboratory time constraints and staffing shortages. In addition to current clinical signs and symptoms, history of sickle cell disease helps participants indicate the need for a transfusion. In the setting of low blood units, health professional participants consistently prioritize children and pregnant mothers, while patients with terminal illnesses such as cancer and the elderly are considered the lowest priority. Many health professionals added that clinical severity played a role in situations where age and outcome were similar between patients, giving priority to those who are more clinically severe. In circumstances where there is no blood available for transfusion at SRRH—a frequent occurrence—health professionals refer patients to MRRH where the regional blood bank is housed. However, several health professional participants noted that patients regularly did not have the funds necessary to travel to MRRH and therefore had to wait for units to become available at SRRH.

### Blood Transfusion Records

Over a period of 46 days, 373 blood transfusions were recorded at SRRH (Table 7). Of these, 55.8% of blood units were allocated to female patients and 33.2% were allocated to children under five years old, comprising the largest age group receiving blood transfusions.

**Table 7:** Characteristics of patients receiving blood transfusions.

Characteristic	Number of Blood Transfusions	Percentage (%)

Sex		
Female	208	55.76
Male	165	44.24
Age (years)		
≤5	124	33.24
6–10	77	20.64
11–20	53	14.21
21–30	48	12.87
31–50	46	12.33
≥51	25	6.70
Blood Group		
A	112	30.03
B	82	21.98
AB	20	5.36
O	159	42.63

### Process Mapping

**Figure 1:**
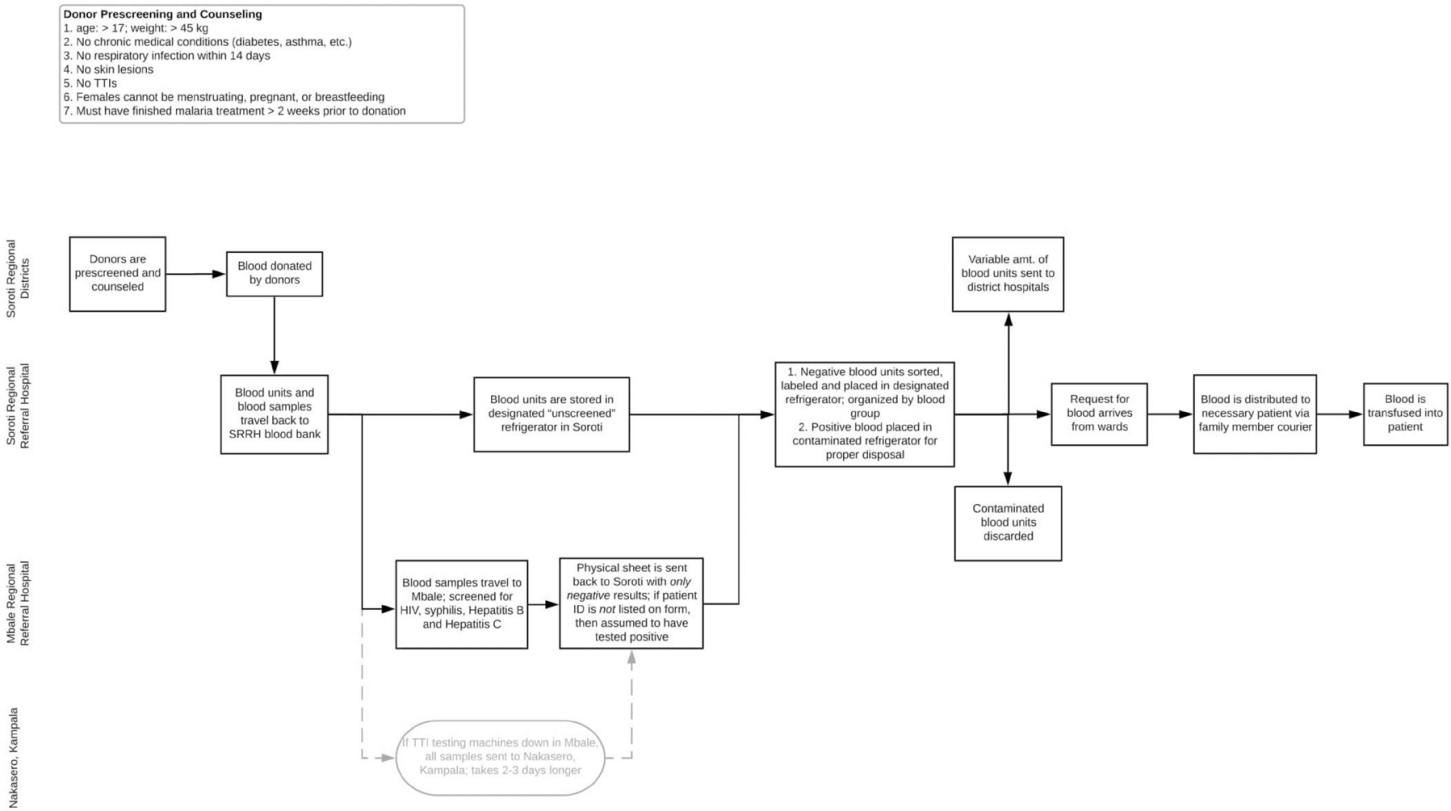
Process map of a unit of blood from donation to transfusion.

Figure 1 details the path of a single unit of blood from time of procurement to transfusion. The details on how a physician determines the need for a blood transfusion have been kept out. Blood units are generally procured outside of the hospital and then transported back to SRRH, from where samples are sent to a TTI testing facility. Blood units are ready to be transfused once results of the TTI testing have returned and contaminated blood units are discarded.

## Discussion

Blood and blood products are life-saving resources in settings of medical illness, obstetric emergencies, trauma, and surgery. However, LMICs often fall short of the blood units needed to satisfy demand [2].

Historically, many LICs have relied on family/replacement or paid blood donors, but this has been slowly decreasing as the push for VNRBD increases. Between 2008 and 2013, the WHO African Region increased VNRBD by 37% [10]. While Africa as a whole continues to have approximately one quarter of blood donations coming from replacement or paid donors, blood units used for transfusions in Uganda are acquired mainly through VNRBD [11]. A 2016 WHO report details age stratification based on country income, with LICs having the highest rate of donation in children less than 18 years old [10]. While secondary school children have historically been targeted due their perceived relative safety from TTIs and consistent location, our research suggests that soliciting blood from the general population may be a beneficial strategy [6]. Studies have shown that general populations in Africa have variable rates of TTIs depending on location, ranging from low (1.3%) in Namibia to high (15.9%) in Dar Es Salaam, Tanzania [12,13]. Ascertaining the TTI rate of community members in Eastern Uganda is crucial in determining how effective soliciting blood from the general population would be with regards to transmissible infections.

Simultaneously, increased outreach and educational programs geared towards the general population of Soroti will be necessary to promote community donation. Concurrent with other studies, a higher level of education (tertiary level) correlated with increased knowledge of blood donation [7]. Surprisingly though, being a student was associated with receiving a lower percentage of knowledge questions correct compared to the rest of the population, a finding that was not reproducible from past literature. Possible reasons why may include lack of education during the donation process and decreased lifetime exposure to blood donation. Interestingly, a study of college students in Ethiopia found a positive correlation between higher levels of family education and increased knowledge of blood donation, highlighting the influence of family members on social activities in close, tight-knit communities [14].

Similar to two studies performed in Northwest Ethiopia, the blood donation rate in Soroti was lower than the prevalence of a positive attitude towards the practice of donating [7,15]. The main deterrents to donating blood cited from participants in our study were lack of food security and sickness—ideas that reflect current economic struggles rather than personal fear surrounding the act of donating blood (i.e. adverse effects, pain from needles) as reported by Asamoah-Akuoko et al. [5] While some of these beliefs are substantiated, such as being HIV positive and not being able to donate blood, others are common misconceptions. For example, having to eat essential fruits and vegetables post-donation, while ideal, is not required; this misunderstanding could easily be dispelled with appropriate education about blood donation. Also, while outreach programs in the community have been the primary means of obtaining blood donations in Uganda, having an easily accessible blood donation site at the hospital may allow for consistent onsite donations by community members. SRRH is an important meeting place in Soroti, both because of its central location and its function as a hospital, and this could be used to the advantage of the blood bank.

Other countries in Sub-Saharan Africa also face challenges maintaining adequate supplies of blood. A 2017 review of the Nigerian blood donation system addressed the widespread difficulty in attaining VNRBD and high rate of TTIs amongst donations [16]. Additionally, multiple articles have emphasized the increased financial burden of maintaining an exclusively VNRBD supply [17,18]. While this is the suggested global standard, some argue that it may be clinically inappropriate to eliminate replacement or paid donors given the severe gap between supply and demand in African countries.

In addition to improving community elements, hospital factors present numerous obstacles to ensure that units obtained in the field are able to be screened and stored appropriately prior to transfusion. The largest bottleneck seen both in the health professional interviews and process mapping focused on the issue of TTI testing. We found that blood unit samples must be transported to Mbale, and often due to malfunctioning machines, travel even further to Nakasero, Kampala, causing a significant delay in TTI results at SRRH, rendering units unavailable for transfusion.

TTI testing is a necessary, but an often time-consuming and expensive process in the setting of complex machinery, lack of technical support, and challenging locale. While advancements of rapid HIV testing over the past decades has created point-of-care testing that is sensitive and accessible, similarly inexpensive testing modalities for the other required TTIs remain elusive in hospitals outside of large, urban cities [19]. For example, nucleic acid testing is used in HICs for TTI detection and is able to identify viral infections in the blood earlier with greater sensitivity, but these remain inaccessible and unaffordable for LICs [20]. Efforts towards incorporating rapid diagnostic testing (RDT) in lieu of older methods such as ELISA and western blots have been increasing [21]. RDTs have been shown to match and increasingly surpass the sensitivity of these older techniques, although certain clinical situations, such as being HIV positive, may decrease sensitivity of the RDTs [22]. However, the lower cost RDTs often are the lowest performing ones, again highlighting the disparity between ability to afford accurate testing and safe transfusions.

Over two million people rely on SRRH, and to minimize delays and maximize treatment for the surrounding population, SRRH needs to have the ability to test for TTIs locally. It is nonoptimal to have blood units sit idly in the blood bank unable to be used because of prolonged TTI testing. Uganda has seven centralized blood banks for a population of almost 43 million people; this creates a significant burden for local hospitals that continue to serve a large portion of the population, but must rely on regional blood banks hundreds of kilometers away. A 2017 article importantly highlights the challenges faced when countries solely depend on centralized blood banking including financial and logistic barriers. The authors suggest that a hybrid model should be used to balance the needs of local hospitals while recognizing the importance of centralized infrastructure [18]. However, machinery and maintenance for TTI testing is costly and therefore a large barrier for SRRH, a hospital with constrained resources, to overcome. Further research regarding low-cost, high-quality TTI testing needs to be undertaken to ensure that the appropriate technology suitable for this locale is invested in.

This study has a number of limitations as it was conducted within a short, eight-week period in Soroti, which is itself an important limitation of the study. First, while we attempted to sample as many different sectors of the population as possible in our attempt to represent the general population of Soroti District, this was done through convenience sampling at different access points (including, as mentioned above, schools, businesses, the market, and farming villages). We did not conduct a formal population-based survey and therefore our findings about community members’ knowledge and attitudes towards blood donation and transfusion should not be interpreted as being fully representative of the population of Soroti District. Second, though the cutoff for whether a community member was “knowledgeable” about blood donation and transfusion (>50% of questions answered correctly) is subjective, we used this cutoff to remain consistent with the study in Gondar Town, Ethiopia. Moreover, the questionnaire we used to assess whether community members could be considered knowledgeable about blood donation has not undergone validation. However, we adapted the questionnaire based on the feedback of collaborators in Soroti. Finally, as with all qualitative studies, interviewees responses’ may have been subject to various biases such as the desire to be socially acceptable and impaired recall. The questionnaires for the study were designed and administered to help minimize the effects of these biases.

The process of collecting blood and preparing it for transfusion is complex and low-resource settings have unique challenges. Inclusion and education of the general population in blood donation outreach campaigns may be a practical intervention to increasing the blood supply at SRRH. Moreover, it is crucial that SRRH has adequate access to TTI testing. Utilizing HIC approaches to maintain an adequate blood unit supply in these settings would be inappropriate, but with a multi-factorial approach both within the community and hospital, there is optimism towards reducing the burden created from the perceived lack of blood at SRRH.
